# Multiple Oncocytic Cystadenoma With Intraluminal Crystalloids in Parotid Gland: Case Report

**DOI:** 10.1097/MD.0000000000000246

**Published:** 2014-12-12

**Authors:** Kayhan Başak, Kumru Kıroğlu

**Affiliations:** From the Department of Pathology, Dr. Lütfi Kırdar Kartal Education and Research Hospital, Istanbul (KB); and Department of Pathology, Kırklareli State Hospital, Kırklareli, Turkey (KK)

## Abstract

Oncocytic cystadenoma is a benign tumor of salivary glands, histologically characterized by multicystic growth of the oncocytic epithelial lining. Crystals in different shapes and nature associate oncocytic type of salivary gland neoplasms.

An 82 year-old woman with right parotideal mass had an operation of superficial parotidectomy. Histological examination revealed multiple unilocular or multilocular cystic lesions with incomplete fibrous capsule, papillary foldings, and 1 or 2 layers of oncocytic epithelium lines. The epithelium lining the cysts were positive for CK8, CK14, CK18, CK19, and negative for SMA, S-100, and p63 immunohistochemically.

Cystadenomas were described as mostly multilocular and we presented a multifocal cystic neoplastic lesion lined by oncocytic type epithelial cells with intraluminal crystalloids. Multiple cysts forming morphology, incomplete fibrous capsule of most cysts and immunohistocemical findings were considered as multiple oncocytic cystadenoma with intraluminal crystalloids in the parotid gland.

## INTRODUCTION

Oncocytic cystadenoma is a rare benign tumor of major salivary glands and histologically characterized by multicystic growth, an oncocytic epithelial lining, which rarely presents with intraluminal crystalloids.^1,2^ Several types of crystalline structures have been documented in association with salivary gland lesions.^3^ The occurrence of multiple oncocytic cysts in the salivary glands with intraluminal crystalloids has been reported only once.^4^ Here we report a case of multiple oncocytic cysts in the parotid gland consistent with neoplastic nature as an oncocytic cystadenoma with intraluminal crystalloids.

## CASE

**FIGURE 1:**
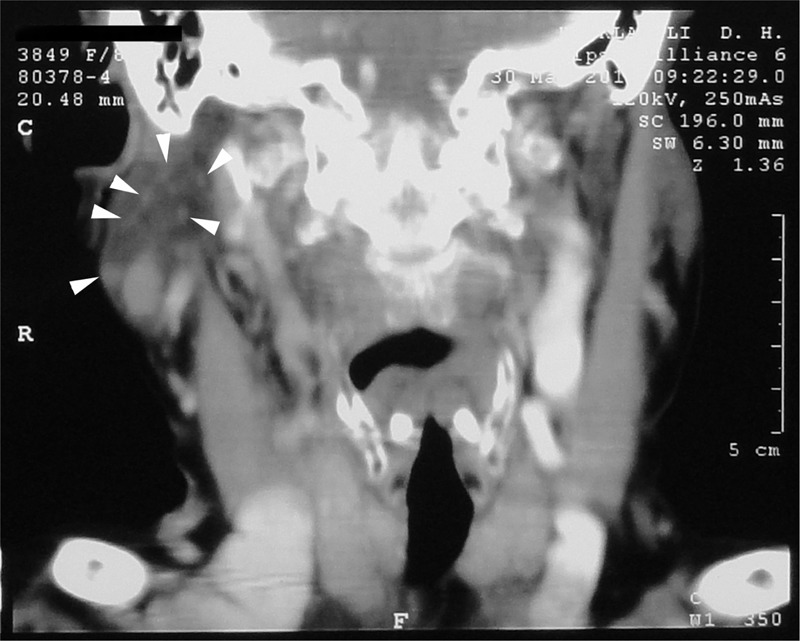
Radiologically multiple microcystic lesions (arrow heads) on right parotideal gland.

**FIGURE 2:**
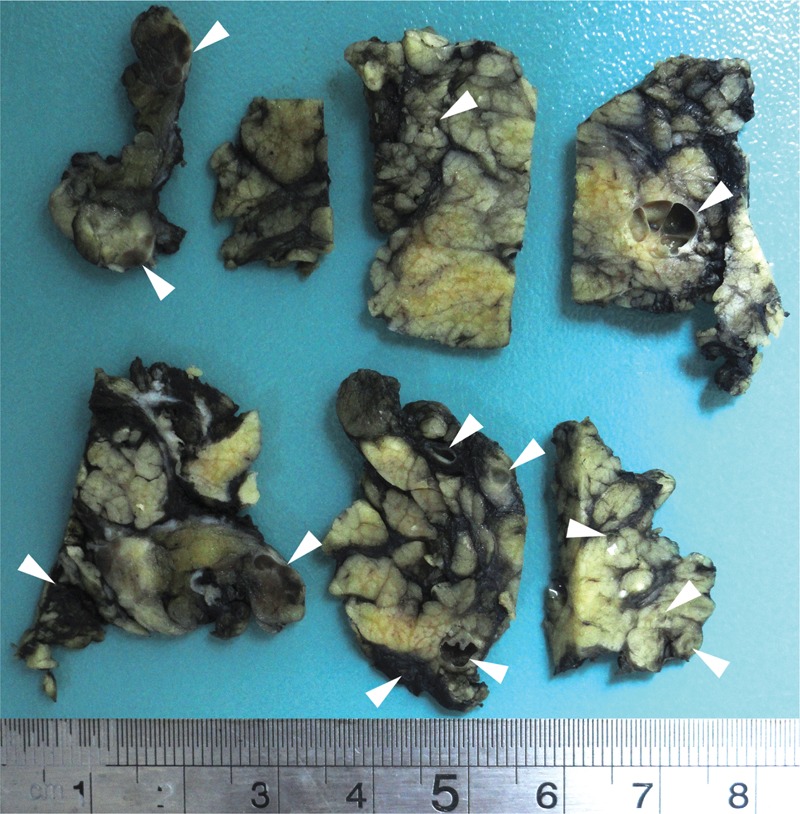
Macroscopically multiple cysts on cut surface of parotis (arrow heads).

An 82-year-old woman was presented with an enlarging lump in the right parotideal region. Medical history included arterial hypertension. On clinical examination, non-tender and softly palpated enlargement of the right parotid gland, with normal appearing overlying skin was observed. No lymphadenopathy was palpated. Radiologically, the neck showed well-circumscribed hypodense lesion measured as 1.8 × 0.8 cm and multiple microcystic lesions inside of the right parotideal gland region (Figure 1). A fine-needle aspiration was performed from the lesion. The cytologic examination showed small groups of oncocytic cells, which were compatible with a benign salivary gland neoplasm. The tumor was surgically removed by superficial parotidectomy. On gross examination, the cut surfaces showed multiple cysts filled with white-colored material, measuring from 0.1 to 0.7 cm in diameter with smooth inner walls (Figure 2). The specimen was fixed in 10% buffered formalin, embedded in paraffin, and 5 μm thick sections were stained with hematoxyline and eosin.

**FIGURE 3:**
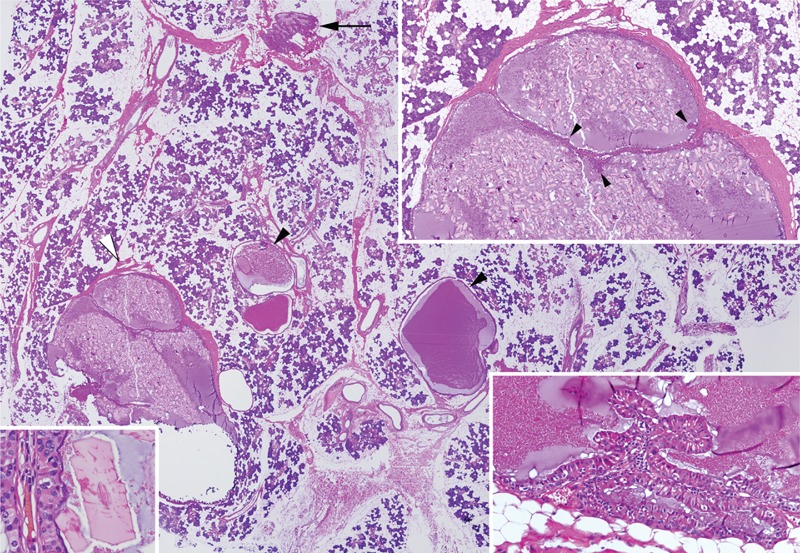
Multiple cysts in normal parotid tissue, some cysts have incomplete fibrous capsule (white arrow head), 1 cyst have papillary foldings (arrow) (whole section, H&E, original magnification 40×). Right upper corner; multilocular cyst with small papillary foldings (arrow heads) and intraluminal crystalloids (H&E, original magnification 40×), right lower corner; papillar foldings lined with double layer cells (H&E, original magnification 400×), left lower corner; intracystic septal wall of one of the multicystic lesion. Double layered oncocytic epithelium and polyhedral crystals (H&E, original magnification 1000×).

Microscopic examination showed multiple separate cysts, dispersed throughout and surrounded by normal parotid tissue with incomplete fibrous capsule. The epithelial lining of the cysts was composed of 1 or 2 layers of columnar to cuboidal oncocytic cells. The lumen of cysts were filled with amorphous secretory material and numerous needle shape and polyhedral glassy pink crystalloids varied in size and shape. There were few papillary folds in some of the cysts (Figure 3). There were not any solid growing areas, cytologic atypia, fibrosis, and lymphoid tissue. Mitoses were very rare.

Immunohistochemical studies were performed using the Bond polymer refine detection method (Leica Biosystems Newcastle Ltd., Newcastle) with diaminobenzidine as the chromogen and hematoxyline as the nuclear counterstain. All immunohistochemical processes were performed using the Leica BOND-MAX automated system (Leica Biosystems, Melbourne). Included antibodies were against CK7 (*clone* OV-TL, 1/300 dilution; Cell Marque, CA), CK8 (*clone* TS1, 1/300 dilution; Thermo Scientific, CA), CK14 (*clone* SPM 263, Ready to use; Thermo Scientific), CK18 (*clone* DC10, 1/100 dilution; Thermo Scientific), CK19 (*clone* b170, 1/200 dilution; Spring, Bioscience, CA), CK20 (*clone* Ks20.8, 1/100 dilution; Thermo Scientific), EMA (*clone* GP1.4, 1/400 dilution; Thermo Scientific), HMWCK (*clone* 34βE12, 1/300 dilution; Genemed, CA), LMWCK (*clone* AE1, Ready to use; Thermo Scientific), Muc-5AC (*clone* CLH2, 1:50 dilution; Leica Biosystems Newcastle Ltd.), CEA (*clone* CD66e Ab-2, 1/500 dilution; Thermo Scientific), GCDFP-15 (*clone* 23A3, 1/100 dilution; Cell Marque), SMA (*clone* 1A4, 1/300 dilution; Bio Care, CA), S-100 (*clone* 4C4.9, Ready to use; SycTech, UT), p63 (*clone* BC4A4, 1/100 dilution; Bio Care), p53 (*clone* DO-7+BP53–12, 1/100 dilution; Lab Vision, CA), DOG1 (*clone* DOG1.1, 1/100 dilution; Bio Care), and Ki67 (*clone* SP6, 1/100 dilution; Bio Care). All antibodies were diluted with Lab Vision™ Antibody Diluent (TA-125-AD, Thermo Scientific™).

**FIGURE 4:**
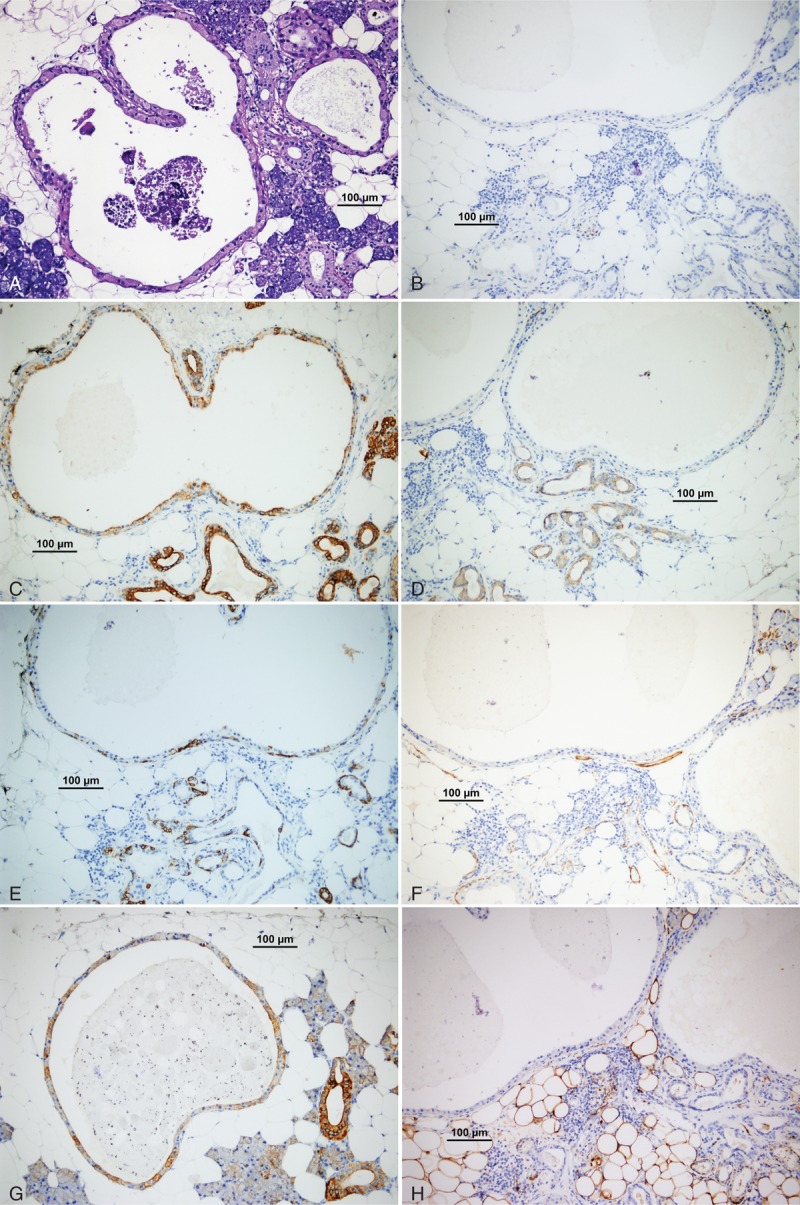
Cysts have no mucinous material with PAS-Ab pH 2.5 stain (A) (PAS-Ab pH 2.5, 100×, bar; 100 μm), cystic epithelium positive for CK8 (B) (CK8, 100×, bar; 100 μm), CK14 (E) (CK14, 100×, bar; 100 μm), and CK18 (G) (CK18, 100×, bar; 100 μm), negative for p63 (B) (p63, 100×, bar; 100 μm), HMWCK (D) (HMWCK, 100×, bar; 100 μm), SMA (F) (SMA, 100×, bar; 100 μm), and S-100 (H) (S-100, 100×, bar; 100 μm).

Epithelium of the cysts were positive for CK7, CK8, CK14, CK18, CK19, LMWCK, EMA (Figures 4), and negative for CK20, HMWCK, Muc-5AC, SMA, S-100, p53, p63, CEA, and DOG1 (Figure 4). Ki67 proliferation index was under 1.0%. There were not any crystals within the stroma or adjacent salivary gland tissue. Crystals were not stained with periodic acid Schiff-Alcian blue pH 2.5 combined stain and Masson Trichrome stain.

## DISCUSSION

Cystadenoma was described as a tumor composed of individual cystic spaces, which are variable in size, commonly multilocular and separated by limited amounts of intervening stroma. Oncocytic variant of cystadenoma was composed predominantly of oncocytes in unilayered or bilayered papillary structures without solid growth.^1–3^ Crystalloids have been shown in a variety of salivary gland tumors and also reported to be found in salivary duct cysts of parotid gland and oncocytic salivary gland neoplasms.^1–4^

In this case, most of cysts have incomplete fibrous capsules and some of cysts have papillary projects with thin fibrovasculary cores. Most of cysts lined by 1 or 2 layers of low columnar oncocytic epithelium. Only 1 cyst was lined explicit double layer oncocytic epithelium. Some cysts were multilocular. Histological pattern was repetitive for multiple cysts regarded as multiple cystadenoma with oncocytic feature. Thirty months after the right superficial parotidectomy, the patient has no clinical complaints and radiological evidence at the left parotid gland or other major salivary glands. Chaushu et al reported a case of multiple oncocytic cyst with crystalloids in parotid gland in a 67-year-old woman having arterial hypertension, likely the case that was presented. The multiple cystic nature of the lesion presumed that the nature of multiple oncocytic cysts with crystalloids in the parotid was a hyperplastic process without neoplastic implications.^4^ In oncocytic cystadenoma of the parotid gland, crystals were identified intraluminally,^1,3,4^ which were shown to be morphologically different from the tyrosine-rich crystalloids and collageneous crystalloids of the extracellular matrix of pleomorphic adenomas and other salivary gland tumors.^1^
